# Fontan-Associated Liver Disease: Predictors of Elevated Liver Stiffness and the Role of Transient Elastography in Long-Term Follow-Up

**DOI:** 10.7759/cureus.85336

**Published:** 2025-06-04

**Authors:** Haruka Sakae, Shiroh Tanoue, Seiichi Mawatari, Kohei Oda, Ohki Taniyama, Ai Toyodome, Sho Ijuin, Kazuaki Tabu, Kotaro Kumagai, Akio Ido

**Affiliations:** 1 Digestive and Lifestyle Diseases, Field of Human and Environmental Sciences, Course of Health Research, Graduate School of Medical and Dental Sciences, Kagoshima University, Kagoshima, JPN; 2 Department of Epidemiology and Preventive Medicine, Field of Human and Environmental Sciences, Course of Health Research, Graduate School of Medical and Dental Sciences, Kagoshima University, Kagoshima, JPN

**Keywords:** congenital heart disease, fontan-associated liver disease, liver stiffness measurement, non-invasive liver fibrosis assessment, transient elastography

## Abstract

Aim: Fontan-associated liver disease (FALD) is common after the Fontan procedure; however, no established non-invasive screening method exists. We aimed to evaluate the utility of transient elastography (TE) for FALD and identify clinical predictors of increased liver stiffness in post-Fontan patients.

Methods: This was a single-center retrospective study of 37 adult patients (≥18 years) who had undergone Fontan procedure and received TE during routine follow-up between February 2019 and June 2024 at Kagoshima University Hospital in Kagoshima, Japan. Clinical, laboratory, cardiac, and abdominal ultrasound data were collected. TE using Fibroscan^®^ was performed by a trained clinician. Reliability criteria included 10 validated measurements and an interquartile range/median of less than 0.3 of the obtained liver stiffness measurements (LSMs). Univariate and multivariate linear regression analyses were performed to identify independent predictors of elevated liver stiffness. Since no validated cut-off values for LSM have been established, LSM was treated as a continuous variable, and exploratory analyses were conducted.

Results: The median age was 27 (19-38) years, with 68% men. The median age at Fontan procedure was four years, and the median duration since Fontan procedure (Fontan duration) was 23 (16-31) years. The median LSM was 13.6 kPa (range: 6.5-38.8), indicating advanced fibrosis or liver cirrhosis. In the univariate analysis, age (B = 0.491, 95% CI: 0.096-0.887, p = 0.016), age at Fontan procedure (B = 1.276, 95% CI: 0.251-2.300, p = 0.016), body mass index (B = 0.833, 95% CI: 0.167-1.499, p = 0.016), B-type natriuretic peptide (BNP) (B = 0.114, 95% CI: 0.002-0.227, p = 0.045), gamma-glutamyl transferase (GGT) (B = 0.042, 95% CI: 0.001-0.083, p = 0.046), fibrosis-4 (FIB-4) index (B = 7.746, 95% CI: 3.212-12.280, p < 0.001), AST-to-platelet ratio index (APRI) (B = 12.873, 95% CI: 4.107-21.639, p = 0.005), systematic ventricular morphology (B = 4.351, 95% CI: 0.747-7.954, p = 0.019), and spleen index (B = 0.363, 95% CI: 0.024-0.702, p = 0.037) were significantly associated with LSM. Variables without missing data were prioritized for inclusion in the multivariate analysis. GGT and BNP were included in the multivariate model as potential confounders, given their established association with both liver congestion. In addition, since FIB-4 had a lower p-value than APRI in the univariate analysis and is considered a more clinically relevant fibrosis marker, it was included in the multivariate analysis. In the multiple regression analysis, the FIB-4 index (B = 6.246, 95% CI: 1.735-10.757, p = 0.008), GGT (B = 0.043, 95% CI: 0.003-0.082, p = 0.034), and age at Fontan procedure (B = 1.141, 95% CI: 0.185-2.097, p = 0.021) were all independently associated with higher LSM.

Conclusion: In adult patients who had undergone a Fontan procedure, factors such as the FIB-4 index, GGT, and age at Fontan procedure were associated with elevated LSM. These findings suggest that TE may be a useful tool for early detection of hepatic fibrosis in high-risk patients, although further studies are needed to confirm these results.

## Introduction

The Fontan procedure is a functional repair procedure for congenital heart diseases, commonly with single-ventricle physiology [1]. Advances in surgical techniques and perioperative management have significantly reduced the early postoperative mortality, leading to improved long-term outcomes in patients who have undergone the Fontan procedure [2,3]. Fontan circulation, characterized by elevated central venous pressure (CVP) and low cardiac output, exerts widespread effects on multiple organ systems, leading to complications such as heart failure, arrhythmias, thromboembolism, protein-losing enteropathy, plastic bronchitis, liver fibrosis and cirrhosis, kidney dysfunction, and lymphatic abnormalities [4,5]. A growing concern among post-Fontan patients is Fontan-associated liver disease (FALD), which includes progressive fibrosis, cirrhosis, and an increased risk of hepatocellular carcinoma (HCC) at a relatively young age [6,7]. A retrospective cohort study reported that only 57% of patients remained cirrhosis-free 30 years after the Fontan procedure [8]. Patients who develop cirrhosis or HCC following the Fontan procedure face a poor prognosis, with studies reporting cumulative 12-month HCC survival rates of 50% [9]. Given this poor prognosis, there is an urgent need to establish standardized methods for monitoring FALD. In FALD, hepatic congestion due to increased systemic venous pressure and reduced oxygen delivery to centrilobular cells leads to centrilobular hepatocyte atrophy, sinusoidal fibrosis, and, eventually, bridging fibrosis, which can progress to cardiac cirrhosis [6]. Owing to the complexity of this pathology, assessing the severity of FALD remains a challenging task. In patients with FALD, conventional serological liver function tests correlate poorly with the degree of fibrosis [8,10]. Although liver biopsy remains the gold standard for assessing fibrosis, it is often impractical due to underlying coagulopathy and the unique hemodynamic alterations associated with Fontan physiology [11]. Thus, liver biopsy is not suitable as a general screening or follow-up method, particularly in patients with FALD. Consequently, no widely accepted non-invasive screening strategy for FALD has been established [12].

In recent years, non-invasive tools have been developed to assess liver fibrosis in chronic liver disease [13-15]. Among the available non-invasive tests for diagnosing and staging liver fibrosis, various methods are employed to measure liver stiffness, including vibration-controlled transient elastography (TE), point shear wave elastography (SWE), two-dimensional SWE, and magnetic resonance elastography (MRE). FibroScan^®^ (a standalone bedside TE device) is a safer and more acceptable non-invasive model for assessing liver fibrosis [13,16]. MRE is also known to provide higher diagnostic accuracy for liver fibrosis, but FibroScan is easier to implement in routine clinical practice, especially in outpatient settings, due to its portability, speed, and lower cost compared to MRE [16,17]. However, the utility of liver stiffness measurement (LSM) in Fontan patients remains uncertain, as it may be confounded by hepatic congestion, potentially leading to an overestimation of fibrosis [18,19]. Nevertheless, emerging evidence suggests that elevated LSM correlates with advanced fibrosis in post-Fontan patients, indicating a potential role for LSM in monitoring disease progression [19-21]. Although predictors of elevated LSM have been suggested, including Fontan duration [22-25], no established predictors of elevated LSM currently exist, and the role of TE in long-term monitoring remains unclear. Furthermore, the development pattern of fibrosis in FALD, which differs from that of other chronic liver diseases, highlights the need for predictive markers of FALD progression beyond LSM alone. Patients with severe FALD are at significant risk of HCC, making early surveillance crucial for facilitating curative interventions that enhance survival rates [26]. Establishing risk-based HCC screening for FALD patients, similar to current practices for prevalent chronic liver diseases and cirrhosis [27], may enable more personalized monitoring based on the severity of liver damage. In this study, we aimed to investigate the clinical factors associated with LSM elevation in post-Fontan patients.

## Materials and methods

Patients

This was a single-center, retrospective cohort study. A total of 46 post-Fontan patients were referred to our department at Kagoshima University Hospital in Kagoshima, Japan, between February 2019 and June 2024. Patients referred to our cardiology department were subsequently seen for FALD screening; all patients who provided consent were included in the study. Patients younger than 18 years (two cases), those with cardiac pacemakers (seven cases), and those with a history of chronic liver disease, including viral hepatitis B or C infection, were excluded. The study excluded patients with pacemakers because, at its outset, the safety of FibroScan for individuals with cardiac implantable devices had not yet been verified in Japan. In addition, patients with a body mass index (BMI) greater than 30 kg/m^2^ were excluded, as obesity may lead to inaccurate TE measurements due to technical limitations [28]. A total of 37 adult patients were included in this study. Laboratory tests and cardiac and abdominal ultrasound (AUS) were performed in all patients. All procedures were conducted on the same day, whereas cardiac ultrasound was performed within one year. The diameter of the inferior vena cava (IVC) was measured on echocardiography in all but one patient with anatomic IVC deficiency. No patients had a history of HCC at baseline; however, two developed HCC during the study period. This study was approved by the Institutional Review Board of Kagoshima University Hospital (approval number: 190161, date of approval: March 23, 2020), and written informed consent was obtained from all participants.

Clinical data

Demographic information, including age, sex, age at the time of the Fontan procedure, preoperative anatomy, systemic ventricular morphology, New York Heart Association functional classification, and liver imaging, including AUS, were collected from medical records. Routine laboratory data, including serum B-type natriuretic peptide (BNP), type IV collagen 7S domain, hyaluronic acid, and Mac2-binding protein glycosylation isomer levels, were obtained before AUS and liver elastography. In addition, fibrosis markers, such as the AST-to-platelet ratio index (APRI) [29], fibrosis-4 (FIB-4) index [29], and model for end-stage liver disease excluding INR (MELD XI) score [30], were calculated. The APRI was calculated as (AST/30 of AST) × 100/ platelet count (10⁹/l), and FIB-4 was calculated as (age × AST)/ (platelet count (10⁹/l) × √ALT). The MELD XI score was calculated as 5.11 × In (total bilirubin (mg/dl) + 11.76 × (creatinine (mg/dl)) + 9.44. If the bilirubin or creatinine value was <1.0 mg/dl, it was set to 1.0 mg/dl in the MELD XI score calculation to prevent negative logarithmic values, as the logarithm of 1.0 equals 0.

AUS and TE

AUS and TE were performed on patients in an overnight fasting state by multiple hepatologists with over 10 years of experience, who were blinded to the clinical data. AUS was conducted using an Aplio-i800 ultrasound system (Canon Medical Systems, Tochigi, Japan), with a 3.5 MHz convex probe. LSM was performed using the Fibroscan 530 Compact device (M-probe, FibroScan®; Echosens, Paris, France). Since there were no obese patients with a high BMI, adjustments to probe size were not necessary. During the examination, patients were positioned in the supine position with the right arm extended to the maximum extent possible to increase the intercostal space. In patients with abdominal situs inversus, the M-probe was placed in the left intercostal space [31]. LSM was excluded if ascites was present on the liver surface. LSM was measured 10 times at the same location using a single probe. The criteria for reliability were 10 validated measurements and a value of less than 0.3 of the interquartile range or median of the obtained liver stiffness values [32]. Liver cirrhosis was diagnosed by a single operator based on ultrasound findings, such as a rounded hepatic limbus, an irregular or nodular liver surface, rough internal structure, and splenomegaly [33,34], or by CT or MRI imaging interpreted by a radiologist. Spleen size was measured in 32 patients in the Fontan group, excluding five patients with asplenia syndrome. The splenic index was calculated from the maximum cross-sectional image of the spleen obtained via intercostal scanning and was defined as the product of the distance between the hilar notch and the anterior cranial end (A cm) and the perpendicular length to this line (B cm): spleen index = 𝐴 × 𝐵 (cm^2^). A spleen index >20 cm^2^ indicated splenomegaly [35].

Statistical analysis　

Categorical data are presented as numbers (n) and percentages (%), while continuous data are expressed as medians and ranges. Since no validated cut-off values for LSM have been established, LSM was treated as a continuous variable, and exploratory analyses were conducted. Factors associated with elevated LSM were evaluated using linear regression analysis, followed by multiple regression analysis employing the forced entry method for variables with a p-value of less than 0.05 in the univariate model. Elevated gamma-glutamyl transferase (GGT) was reported as the most common abnormality observed in post-Fontan patients [36]. Therefore, we included GGT in the analysis. In addition, liver function tests, splenomegaly, and liver fibrosis factors, including the FIB-4 index, were included in the analysis as they are widely recognized as non-invasive markers that reflect the severity of liver disease and may contribute to increased LSM in patients with FALD.

Although linear regression was conducted under the assumption of normality, Spearman’s rank correlation was also performed as a non-parametric sensitivity analysis to account for the small sample size and to confirm the reliability of the association. Variables with a variance inflation factor >10 were excluded to address multicollinearity. Statistical significance was set at p < 0.05. All statistical analyses were performed using IBM SPSS Statistics for Windows, Version 27.0 (released 2019, IBM Corp., Armonk, NY).

## Results

Patients

The baseline characteristics of the patients who underwent the Fontan procedure are presented in Table 1. This cohort consisted of 37 patients who were prospectively evaluated using a multimodal approach. The median age of the patients was 27 years, and 68% were male. The median age at the Fontan procedure was four (1-11) years. The proportion of systemic right ventricle, left ventricle, and both ventricle predominance was 54%, 41%, and 5%, respectively. Three patients reported a history of protein-losing gastroenteropathy. Thirty-five patients (95%) were administered oral anticoagulants. Further hepatic assessment and echocardiography results are shown in Table 2. Aminotransferase, cholinesterase, total bilirubin, albumin, and alpha-fetoprotein levels were within the normal range, whereas the GGT level (64 U/mL) was increased slightly. Two patients (5%) exhibited elevated alpha-fetoprotein levels and were subsequently diagnosed with HCC. Echocardiography revealed a median ejection fraction of 53%. Abdominal imaging revealed cirrhotic patterns in nine (24%) patients. The median LSM was 13.6 kPa.

**Table 1:** Patient characteristics Data reported as median (range) or count (% of total). Abbreviations: NYHA, New York Heart Association *Indeterminate: Cases in which ventricular dominance could not be determined, despite available imaging and surgical data, because of unclear or inconclusive morphological features.

Characteristics (n = 37)	Median (range)
Age (years)	27 (19–38)
Sex (men/women)	25 (68%)/ 12
Body mass index	20.2 (15.9–29.7)
Age at the Fontan procedure (years)	4 (1–11)
Systemic ventricle* (right/ left/ indeterminate)	20 (54%)/ 15/ 2
Fontan duration (years)	23 (16–31)
NYHA classification I/Ⅱ	7/ 30 (81%)
Situs inversus	3 (8%)
Asplenia	5 (14%)
Protein-losing enteropathy	3 (8%)
Medication	
Oral anticoagulants	35 (95%)
Diuretics	10 (27%)
Pulmonary vasodilators	5 (14%)

**Table 2:** Results of hepatic assessment and echocardiography Data were reported as median (range) or count (% of total). Abbreviations: Alb, albumin; AST, aspartate aminotransferase; ALT, alanine aminotransferase; GGT, gamma-glutamyl transferase; ChE, cholinesterase; AFP, alpha-fetoprotein; BNP, brain natriuretic peptide; T4C7S, type IV collagen 7S; HA, hyaluronic acid; M2BPGi, Mac-2 binding protein glycosylation isomer; FIB-4 Index, Fibrosis-4 Index; APRI, AST-to-platelet ratio index; LSM, liver stiffness measurement; EF, ejection fraction; IVC, inferior vena cava †Missing data for splenomegaly (n = 5, 14%) due to asplenia and missing data for the maximum IVC diameter (n = 1, 3%) due to IVC deficiency.

Characteristics (n = 37)	Median (range)
Laboratory data	
Platelet count (10^4^/µl)	19.4 (7.8–37.4)
Alb (g/dl)	4.6 (2.5–5.3)
AST (U/l)	24 (11–46)
ALT (U/l)	23 (10–39)
GGT (U/l)	64 (21–248)
Total bilirubin (mg/dl)	1.1 (0.4–2.7)
ChE (U/l)	309 (158–423)
AFP (ng/ml)	2.4 (0.3–37.2)
BNP (pg/ml)	12 (6–93)
T4C7S (≤4.4) (ng/ml)	5.8 (4.0–16.2)
HA (≤50) (ng/ml)	19 (9–88)
M2BPGi (<1.0) (cut-off index)	0.3 (0.1–1.0)
FIB-4 index (<1.3)	0.66 (0.28–2.34)
APRI (<0.5)	0.4 (0.16–1.07)
MELD Ⅺ score (<9.44)	9.92 (9.44–14.82)
Abdominal ultrasound	
Splenomegaly, n (%)^†^(missing data = 5)	17 (46%)
Ascites, n (%)	7 (19%)
Nodules, n (%)	9 (24%)
Abdominal image findings	
Cirrhosis, n (%)	9 (24%)
Elastography	
LSM (kPa)	13.6 (6.5–38.8)
Echocardiography	
EF (%)	53 (32–70)
Maximum IVC diameter (mm) ^†^ (missing data = 1)	15 (7–21)

Association of LSM and different parameters

**Figure 1:**
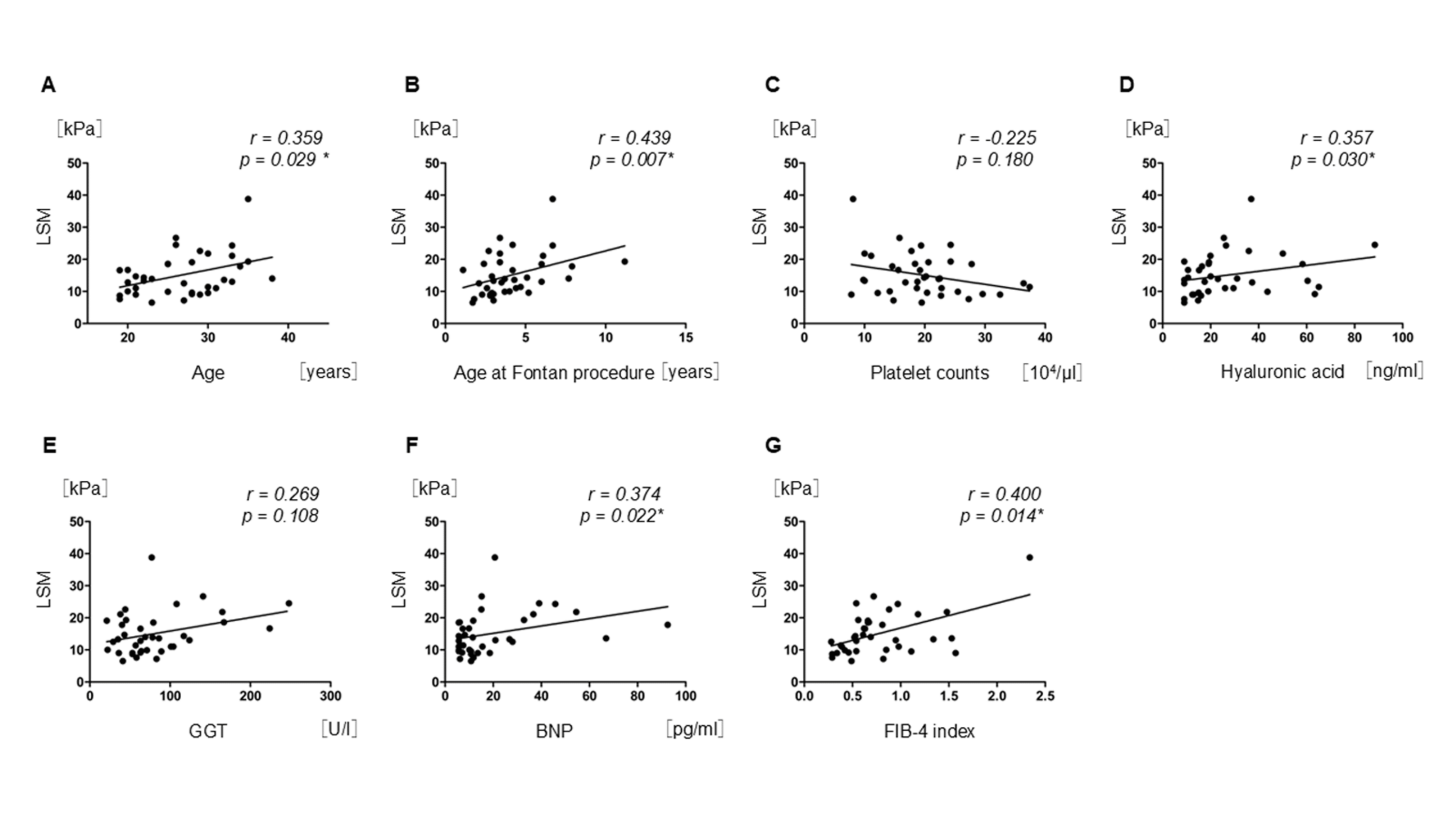
Correlation between LSM and the different parameters Scatter plot showing the correlation between liver stiffness measurements (LSM) and different parameters. Correlations between LSM and age (A), age at the Fontan procedure (B), platelet counts (C), hyaluronic acid (D), GGT (E), BNP (F), and FIB-4 index (G) were tested using Spearman’s rank correlation coefficient. LSM was significantly correlated with age (r = 0.359, 95% CI: 0.030–0.618, p = 0.029), age at the Fontan procedure (r = 0.439, 95% CI: 0.124–0.673, p = 0.007), hyaluronic acid (r = 0.357, 95% CI: 0.028–0.617, p = 0.030), BNP (r = 0.374, 95% CI: 0.047–0.629, p = 0.022), FIB-4 index (r = 0.400, 95% CI: 0.077–0.647, p = 0.014). No significant correlation was found between LSM and platelet counts (r = -0.225, 95% CI: -0.519–0.116, p = 0.180). Abbreviations: LSM, liver stiffness measurement; GGT, gamma-glutamyl transferase; BNP, brain natriuretic hormone; FIB-4 Index, Fibrosis-4 Index

Figure 1 depicts the correlation between LSM and the different parameters. LSM was significantly correlated with age (r = 0.359, 95% CI: 0.030-0.618, p = 0.029), age at the Fontan procedure (r = 0.439, 95% CI: 0.124-0.673, p = 0.007), hyaluronic acid level (r = 0.357, 95% CI: 0.028-0.617, p = 0.030), BNP level (r = 0.374, 95% CI: 0.047-0.629, p = 0.022), and FIB-4 index (r = 0.400, 95% CI: 0.077-0.647, p = 0.014).

Table 3 summarizes the association between LSM and different parameters in the univariate linear regression analysis. Age (B = 0.491, 95% CI: 0.096-0.887, p = 0.016), age at the Fontan procedure (B = 1.276, 95% CI: 0.251-2.300, p = 0.016), BMI (B = 0.833, 95% CI: 0.167-1.499, p = 0.016), BNP level (B = 0.114, 95% CI: 0.002-0.227, p = 0.045), GGT level (B = 0.042, 95% CI: 0.001-0.083, p = 0.046), FIB-4 index (B = 7.746, 95% CI: 3.212-12.280, p < 0.001), APRI (B = 12.873, 95% CI: 4.107-21.639, p = 0.005), systematic ventricular morphology (B = 4.351, 95% CI: 0.747-7.954, p = 0.019), and spleen index (B = 0.363, 95% CI: 0.024-0.702, p = 0.037) were significantly related with LSM in the univariate analysis. Despite the absence of a statistically significant difference, a trend toward a negative correlation was observed between LSM and platelet count (B = -0.279, 95% CI: -0.579-0.021, p = 0.067).

**Table 3:** Univariate linear regression analysis for the liver stiffness measurement *B = unstandardized regression coefficient; SE = standard error; CI = confidence interval Data are reported as medians or counts (% of total). Abbreviations: LSM, liver stiffness measurement; NYHA, New York Heart Association; BMI, body mass index; Alb, albumin; AST, aspartate aminotransferase; ALT, alanine aminotransferase; GGT, gamma-glutamyl transferase ChE, cholinesterase; BNP, brain natriuretic hormone; T4C7S, type IV collagen 7S; HA, Hyaluronic acid; M2BPGi, Mac-2 binding protein glycan isomer; FIB-4 Index, Fibrosis-4 Index; APRI, AST-to-platelet ratio index; MELD-XI; Model for Endstage Liver Disease Excluding INR score; EF, ejection fraction; IVC, inferior vena cava; CO, cardiac output; CI, cardiac index * P < 0.05 †Missing data for splenomegaly (n = 5, 14%) due to asplenia, and missing data for the maximum IVC diameter (n = 1, 3%) due to IVC deficiency.

Factors	B	SE	95%CI		p
Age (years)	0.491	0.195	0.096	0.887	0.016*
Sex (men), n (%)	2.369	2.345	-2.392	7.129	0.319
Age at Fontan procedure (years)	1.276	0.505	0.251	2.300	0.016*
Fontan duration (years)	0.469	0.255	-0.049	0.987	0.074
NYHA	-5.411	2.692	-10.877	0.054	0.052
BMI (kg/m^2^)	0.833	0.328	0.167	1.499	0.016*
Systematic ventricular morphology	4.351	1.775	0.747	7.954	0.019*
Laboratory data					
Platelet count (10^4^/µl)	-0.279	0.148	-0.579	0.021	0.067
Alb (g/dl)	-2.009	2.012	-6.094	2.077	0.325
AST (U/l)	0.328	0.169	-0.015	0.670	0.060
ALT (U/l)	0.215	0.141	-0.070	0.501	0.135
GGT (U/l)	0.042	0.020	0.001	0.083	0.046*
Total bilirubin (mg/dl)	2.677	2.068	-1.521	6.874	0.204
ChE (U/l)	0.012	0.017	-0.023	0.047	0.482
BNP (pg/ml)	0.114	0.055	0.002	0.227	0.045*
T4C7S (≤4.4) (ng/ml)	0.545	0.501	-0.472	1.562	0.284
HA (≤50) (ng/ml)	0.092	0.056	-0.021	0.206	0.108
M2BPGi (<1.0) (cut off index)	3.746	6.148	-8.735	16.227	0.546
FIB-4 index (<1.3)	7.746	.2.223	3.212	12.280	<0.001*
APRI (<0.5)	12.873	4.318	4.107	21.639	0.005*
MELD Ⅺ score (<9.44)	0.944	0.689	-0.455	2.343	0.179
Abdominal ultrasound					
Spleen Index (Missing data = 5)^†^	0.363	0.166	0.024	0.702	0.037*
Echocardiography					
EF (%)	-0.092	0.147	-0.391	0.207	0.536
IVC (mm) (Missing data = 1)†	0.221	0.348	-0.485	0.928	0.528
CO (L/min)	-0.090	0.649	-1.412	1.233	0.891
CI (L/min/m^2^)	-0.878	1.338	-3.607	1.852	0.517

Table 4 summarizes the association between LSM and different parameters in the multiple regression analysis. Variables without missing data were prioritized for inclusion in the multivariate analysis. Among the variables that showed significance in the univariate analysis, sex and age at the Fontan procedure were included. GGT and BNP were included in the multivariate model as potential confounders, given their established association with liver congestion. The model was adjusted for sex, age at the Fontan procedure, GGT, BNP, and FIB-4 index. Age at the Fontan procedure (B = 1.141, 95% CI: 0.185-2.097, p = 0.021), GGT level (B = 0.043, 95% CI: 0.003-0.082, p = 0.034), and FIB-4 index (B = 6.246, 95% CI: 1.735-10.757, p = 0.008) were significantly related to LSM in the multiple regression analysis.

**Table 4:** Multivariate regression analysis for the liver stiffness measurement R^2^ = 0.466, adjusted R^2^ = 0.380 *B = unstandardized regression coefficient; SE = standard error; CI = confidence interval, VIF = variance inflation factor ** Multivariable model adjusted for sex, age at the Fontan procedure, GGT, BNP, and FIB-4 index. Data are reported as medians or counts (% of total). Abbreviations: LSM, liver stiffness measurement; GGT, gamma-glutamyl transferase; BNP, brain natriuretic hormone; FIB-4 Index, Fibrosis-4 Index

Factors	B	SE	95%CI		p	VIF
Sex (men), n (%)	0.517	2.138	-3.843	4.877	0.811	1.340
Age at the Fontan procedure (years)	1.141	0.469	0.185	2.097	0.021*	1.213
GGT (U/l)	0.043	0.019	0.003	0.082	0.034*	1.316
BNP (pg/ml)	0.006	0.054	-0.104	0.117	0.910	1.424
FIB-4 index	6.246	2.212	1.735	10.757	0.008*	1.212

## Discussion

This study aimed to identify the clinical predictors of elevated liver stiffness in patients who have undergone a Fontan procedure. Despite the absence of established LSM cut-offs for Fontan patients, the median LSM was as high as 13.6 kPa (6.5-38.8), which indicated cirrhosis in the biopsy-matching study of chronic hepatitis [32,37]. The cutoff value for LSM related to fibrosis progression in patients after the Fontan procedure has not yet been established. However, previous studies involving both pediatric and adult post-Fontan patients have reported LSM values ranging from 14.6 to 27.6 kPa [22-24,38]. The LSM values in our study were lower than those reported in this data. This may be due to our patients' preserved Fontan circulation, resulting in less congestion and fibrosis. Further cardiac catheterization and liver biopsies are needed to clarify this estimation.

Appropriate strategies for diagnosing and staging FALD remain challenging [9]. In post-Fontan patients, cirrhosis is diagnosed based on a combination of clinical, laboratory, and radiological findings [33]. Liver biopsy is the gold standard for detecting liver fibrosis in FALD; however, it is not suitable for routine longitudinal follow-up because of its invasive nature [39]. Therefore, identifying non-invasive alternatives to diagnose and monitor FALD is necessary [12].

TE is widely used in post-Fontan patients. Although LSM may be confounded by liver congestion in post-Fontan patients, a recent study reported that liver histology showed a good correlation with the FIB-4 index and APRI, and LSM had high sensitivity in adult post-Fontan patients [40]. The FIB-4 index and APRI are important non-invasive markers of fibrosis that can be easily calculated using routine laboratory tests [30]. A cohort study revealed that liver fibrosis was associated with a lower platelet count, higher bilirubin level, and higher FIB-4/APRI scores in post-Fontan patients [41]; higherFIB-4/APRI scores were also associated with long-term all-cause mortality in Fontan patients [42].

Our study revealed that the FIB-4 index, GGT level, and age at the Fontan procedure may be predictors of increased LSM, even in postoperative Fontan patients. Previous reports indicated that LSM was associated with age at the Fontan procedure [22], IVC diameter [22], APRI [22,23], spleen size [22,23], and low platelet count [23]. Our study showed that LSM was not affected by these cardiac parameters when assessing congestion, and LSM was correlated with FIB-4 but not with BNP in the multivariate analysis. Although previous studies have reported an association between APRI and LSM, we observed a correlation between the FIB-4 index and LSM. The relationship between hemodynamic parameters and LSM requires further investigation; however, our study suggests that LSM may be a valuable tool for the early diagnosis of liver fibrosis resulting from FALD.

LSM was also correlated with GGT levels in the multivariate analysis, a finding that has not been previously reported. Elevated GGT levels are a characteristic of congestive hepatopathy [43]. GGT elevation is caused by increased CVP and is associated with poor prognosis in patients with heart failure [44]. A nationwide survey revealed that GGT elevation was the most prevalent abnormality in post-Fontan patients, with 45% of the patients exhibiting elevated GGT levels also presenting with liver fibrosis at the time of FALD diagnosis [36]. A single-center study (n = 52) suggested that elevated GGT levels may be correlated with portal hypertension in patients with FALD [45]. However, elevated GGT levels alone are not specific to fibrosis in post-Fontan patients [46-48]. Kogiso et al. reported that in patients with no improvement in GGT levels over a five-year observation period, the platelet count decreased over time, suggesting progression of fibrosis [49]. This suggests that GGT is a biomarker of FALD progression and that monitoring the long-term course of GGT and LSM may provide complementary information on the progression of fibrosis.

Several reports have documented the factors that contribute to the development of fibrosis in FALD, and the duration of Fontan circulation has consistently been identified as a predictor of fibrosis and cirrhosis [46,50]. LSM is also correlated with Fontan duration [22-25]. However, another study showed that older age at Fontan was associated with a higher histological severity of liver fibrosis [51]. In our study, LSM was correlated with age at Fontan procedure but not with Fontan duration. Elevated LSM is common even early after the Fontan procedure, and LSM increases significantly shortly after the Fontan procedure, indicating that an increased afterload may contribute to elevated LSM [38,52]. These findings suggest that a longer post-Fontan period may be associated with higher LSM values. The association between age at Fontan procedure and LSM suggests that these patients may have developed advanced FALD, highlighting the importance of not missing the timing for the Fontan procedure. Previous cohorts of post-Fontan patients who underwent liver biopsy and cardiac catheterization have demonstrated that age and time since Fontan correlate with collagen deposition; however, significant interpatient variability suggests that Fontan duration is not the only factor associated with hepatic changes [46]. These findings are consistent with our results, which suggest that Fontan duration alone does not fully explain the extent of hepatic changes in this population. However, the absence of this association in our study may also be due to the small sample size. A previous study suggested otherwise, indicating that injury before the Fontan procedure, including prolonged hospitalizations and repeated hypoxic episodes, may contribute to the development of fibrosis [53]. Thus, it is possible that pre-Fontan events contributed to the current state of liver dysfunction, potentially leading to increased liver stiffness in older patients who underwent the Fontan procedure.

The utility of TE is limited by the challenge of distinguishing whether the increased LSM is driven by hepatic congestion or fibrosis. However, changes in imaging findings do not appear until fibrosis progresses [39,54]. Although histological validation is lacking, our study suggests that TE may serve as a valuable tool for assessing FALD progression. Patients with a higher FIB-4 index, elevated GGT levels, and older age at the time of the Fontan procedure may benefit from proactive assessment with TE, as its non-invasive and reproducible nature makes it a valuable tool for early detection and monitoring of hepatic complications. As this study is single-center, cross-sectional, and based on a small cohort, we could not determine definitive guidelines regarding monitoring frequency or action thresholds for TE. Depending on the risk profile for progression of FALD, it may be appropriate to tailor TE monitoring intervals, such as annually for low-risk patients and every six months for high-risk patients with higher FIB4 levels, elevated GGT levels, and a higher age at the Fontan procedure. Further studies are needed to clarify the clinical utility and cost-effectiveness of this risk-based approach.

It remains unclear whether an increase in LSM reflects liver fibrosis or congestion and whether fibrosis is often overestimated. In addition, TE is a non-invasive diagnostic tool that cannot replace liver biopsy. However, because TE is non-invasive and can be measured repeatedly, it is useful for longitudinal follow-up to monitor the worsening of FALD. This allows for monitoring the progression of FALD, a role that is likely to expand with the increasing number of patients following the Fontan procedure. Our results provide valuable insights into the management of FALD. Further large-scale studies with long-term follow-up are required to validate our findings.

This study had some limitations. First, the liver histology and cardiac catheterization findings were unavailable. Second, our study was retrospective and limited to a single tertiary institution. In addition, not all eligible patients were enrolled, which introduces a potential risk of selection bias. Third, in a small number of cases, patients have undergone TE and echocardiography on different days. We believe the impact of timing differences is limited because we utilized data from echocardiography performed within a year, which is a standard practice in routine clinical care. Moreover, although FibroScan measurements were conducted by multiple trained operators, the intra- and interobserver variability was not formally assessed in this study. This may have introduced measurement variability that should be considered when interpreting the results. Overall, the results of this study are preliminary and based on a small, single-center cohort; thus, these results should not be deemed sufficient to recommend routine implementation at this time. Further validation and large-scale, multi-center studies are necessary to confirm these findings before widespread use can be endorsed.

## Conclusions

TE is a non-invasive and easily repeatable test, even in postoperative Fontan patients, making it an ideal tool for the long-term monitoring of liver fibrosis. Patients with a higher FIB-4 index, higher GGT level, and older age at the time of their Fontan procedure are likely to benefit the most from such assessments. These results suggest that TE should be used proactively in such patients to aid in the early detection of liver fibrosis and ensure timely referral to a hepatologist and additional imaging diagnosis. This approach would enhance the follow-up strategy for routine TE and additional imaging in these patients, directing attention to those most likely to benefit from further evaluation.

For future research, we propose developing a protocol, such as the cutoff value of LSM in patients after the Fontan procedure. Further studies are required to establish reliable non-invasive screening methods for assessing fibrosis, including serum biomarkers and imaging modalities. This study was conducted at a single institution and included a small number of patients, which limits its generalizability and does not support its widespread use. Longitudinal or large-scale multi-center studies are needed to confirm these results and develop guidelines.
